# Four-Year-Old's Online Versus Face-to-Face Word Learning via eBooks

**DOI:** 10.3389/fpsyg.2021.610975

**Published:** 2021-03-12

**Authors:** Paola Escudero, Gloria Pino Escobar, Charlotte G. Casey, Kristyn Sommer

**Affiliations:** ^1^The MARCS Institute for Brain, Behaviour and Development, Western Sydney University, Bankstown, NSW, Australia; ^2^Australian Research Council Centre of Excellence for the Dynamics of Language, Australian National University, Canberra, ACT, Australia

**Keywords:** online developmental science, online testing, preschool children, word learning, language acquisition, eBook, Zoom, online methods

## Abstract

Developmental research typically relies on face-to-face testing at laboratories, childcare centers, museums or playgroups. Current social distancing measures have led to a halt in research. Although face-to-face interaction is considered essential for research involving young children, current technology provides viable alternatives. This paper introduces an accessible, replicable and easy to follow method to conduct online developmental research with young children employing a word-learning task as an exemplar, including a detailed workflow and step-by-step guide to using easily accessible programs and platforms. Four-year-old children's (*N* = 56) performance on a word-learning task delivered online vs. face-to-face is provided as a method validation. Children's performance on the word-learning task was predominantly consistent across delivery modes, with only slight variation in performance between modes. The implications of these similar results across face-to-face and online methods are discussed alongside avenues for further research. Importantly, this paper presents an emerging methodological approach for the online administration of developmental science both within and beyond the COVID-19 pandemic, adding a new tool to current and future developmental scientist's toolkits.

## Introduction

The radical measures taken to control the COVID-19 pandemic, including state and country-wide lockdowns and social distancing, have resulted in the interruption of data collection for research that relies on face-to-face interaction. Current expert advice suggests that the risk of infection, including second waves in countries once considered low-risk, and the sanitary policies in place are likely to persist beyond 2022, making face-to-face human research unlikely for the foreseeable future (Gentili and Cristea, 2020; Kissler et al., 2020). Researchers have thus urged for a restructure of research policies and practices to enable large collaborations across labs and the publication of unfinished research (Gentili and Cristea, 2020). Proposals for a move to online testing have been highlighted as vital (Gentili and Cristea, 2020) for the continued reach and impact of developmental research (Sheskin et al., 2020).

Large-scale efforts are currently underway to facilitate large, reproducible developmental research (Sheskin et al., 2020). The framework proposed by Sheskin et al. (2020), dubbed the online Collaboration for Reproducible and Distributed Large-scale Experiments (CRADLE), will further transform the field of developmental science alongside the ManyBabies initiative (Frank et al., 2017). However, for small-scale science at the individual researcher or student level, particularly for those traditionally trained in face-to-face testing, online studies appear an ominous, albeit unavoidable option should they wish to continue producing research. Due to the inherent challenges associated with testing young children online, resources and exemplars for how face-to-face child experiments can be adapted for the online environment are crucial but scarce.

The Child Lab at Yale University has pioneered methods for testing children online, documenting the online adaptation of several seminal developmental studies including a false-belief task, a fairness task, a gravity task and memory tasks in 5- to 12-year-old children (Sheskin and Keil, 2018). Sheskin and Keil (2018) identified that the online administration of these tasks was mostly successful. Yet unexpectedly, the online administration of the false-belief task did not mirror typical face-to-face performance. The “Sally-Anne” false belief task is typically passed by the age of 4 or 5 years when administered face-to-face (Baron-Cohen et al., 1985; Callaghan et al., 2012), however Sheskin and Keil (2018) found that only 64% of 7- to 8-year-olds passed their online version. Given that a direct comparison between online and face-to-face delivery of their task was not conducted, it is difficult to conclude whether delivery mode is the cause of the discrepancy between their findings.

A primary concern for online developmental research, which may have precluded larger scale uptake of online research historically, is that it necessitates greater involvement from a parent, in some instances requiring them to become a protégé research assistant. Without training in experimental protocols or an understanding of the importance of impartial responses, there is a chance that parents may accidentally influence their child's performance. Kapitány et al. (2020) conducted a digital survey which was administered by either a parent in their own home or by a research assistant in a science museum. They found comparable results across both parent and experimenter administered surveys and that with only a brief note at the beginning of the survey to remain impartial parents were capable of administering a survey to their child without influencing their responses. Although online surveys have a smaller margin for potential parental influence than live interactive experiments, this evidence suggests that, with some guidance, parents are capable of assuming the role of research assistant for conducting research via distance.

Another challenge immediately evident in the current efforts for online testing with young children is the need for specialized software not previously encountered by researchers and parents and would require additional training (e.g., https://lookit.mit.edu/). The current study proposes to reimagine developmental research away from the laboratory, museums or childcare centers as a viable and accessible resource, by using (a) technology that is readily available in children's homes, (b) straightforward recruitment and set up protocols for parents, and (c) simple tasks that are easily adaptable for online delivery. Specifically, we have combined, adapted, and extended tools available from previous research (Sheskin and Keil, 2018; Social Learning Lab, 2020) to propose an online testing paradigm that is administered via Zoom (https://zoom.us/), one of the most widely used video conferencing platforms. We use simple presentation slides, that we adapted from those available on the Stanford Social Learning Lab, for parents to set up the Zoom session, together with instructions on their involvement during the session.

The proposed online testing paradigm has the potential to be adapted to a multitude of developmental tasks and measures (e.g., video-based tasks, physical tasks that can be demonstrated to the child and require verbal or gestural responses), and may be implemented with child participants of varying ages [e.g., Escudero et al. (2021) reports a similar paradigm for online testing of adult participants]. In the current study, we focus on replicating a word learning study that we had previously conducted in a face-to-face environment prior to the COVID-19 outbreak [which is reported within a larger study in Pino Escobar et al. (under review)].

Word learning is a crucial developmental skill which requires children to associate a spoken label to its referent, thus integrating their auditory and visual environment into a communicable context (Quine, 1960). Research into word learning therefore is valuable to continue in spite of the current pandemic and we speculate is an important foundational concept for validating the online developmental science methodology. There are a multitude of ways in which word learning has been investigated historically, however, one paradigm that lends itself well to online delivery is word learning from storybooks delivered in the eBook format. Children are found to successfully learn new words from storybook reading both in print (Flack et al., 2018) and digital mediums (i.e., eBooks) (Reich et al., 2016). For eBooks in particular, when they are designed well, preschool-aged children's word learning is equal if not better than from traditional print books (Reich et al., 2016; Etta and Kirkorian, 2019). Thus, for the purposes of extending research into word learning during the COVID-19 pandemic, we will employ a word learning task embedded within an eBook as our validation of the online delivery methodology. We predicted comparable performance between online and face-to-face children's word-learning given recent studies reporting successful learning for similar tasks. Specifically, Gaudreau et al. (2020) demonstrated that 4-year-olds equally comprehended and learned from a story read live face-to-face, live via video chat or through a pre-recorded video and were as responsive in the face-to-face and live video chat conditions. Regarding word learning, Myers et al. (2016) found that 2-year-olds readily learned words from video chat sessions with an experimenter. As well, we hypothesize that despite the potential reduction in engagement, a video chat may yield similar results to live word learning studies because performing a task from the comfort of the child's home may be more conducive for learning than a noisy childcare or an artificial laboratory, particularly for 4-year-olds who are easily distracted.

In the current study, parents and children observed and listened to an eBook story via Zoom and children's memory for the words presented in the story were assessed with a simple picture-choice task. Unlike reports of discrepancies between laboratory and online testing with adults (e.g., Crump et al., 2013), to our knowledge our results provide the first validation of comparable child-based experimental data across face-to-face and online testing modes, with encouraging signs that our online method is not only engaging but successful.

## Materials and Methods

The participant and procedural components of our online study were pre-registered on the Open Science Framework (OSF) (https://bit.ly/2L0S439) as part of a larger study. Further information and documentation can be found in this OSF link and in the Supporting Information. Prior to analyzing the full data set from all the tasks included in the pre-registered study, we chose to first analyze and report on a subset of the data to describe and validate our online method. Thus, this paper presents a comparison of our first task to a pre-existing sample that was collected face-to-face prior to our online study. Here we present a summary of the complete online experimental process and workflow. The procedure from recruitment to testing was conducted virtually and the workflow was adapted from the Social Learning Lab resources (Social Learning Lab, 2020).

### Participants

A total of 41 children participated in this online study (M age = 4.72 years, SD = 0.47 years, range = 4 years, 0 months – 5 years, 3 months; 25 female). Participants were recruited from a university database of parents who had volunteered to participate in child research. Participants were able to schedule their own appointment time and received follow-up emails and reminders using an automatized appointment scheduling program (https://calendly.com/). After a parent agreed to participate, a confirmation email was sent containing a link to the Participant Information Sheet, the Participant Consent Form and a Demographics survey (administered using Qualtrics software, Qualtrics, Provo, UT, USA. https://www.qualtrics.com). Children received an electronic certificate for participation (see Supplementary Figure 1 for a visual illustration of the recruitment workflow).

The 41 children were tested in two groups. Online group 1 included 15 children (M age = 4.9 years, SD = 0.07 years, range = 4 years, 3 months – 5 years, 3 months; 8 female) who were selected based on an age and gender match to a control group of children tested face-to-face to enable direct comparison between online and face-to-face testing. The control face-to-face group consisted of 15 children (M age = 4.9 years, SD = 0.29 years, range = 4 years, 3 months – 5 years, 4 months; 7 female). They were recruited and tested in childcare centers (*N* = 6) and a university laboratory (*N* = 9) in 2019, prior to the COVID-19 outbreak, and their results are reported as part of a larger study which included an additional 32 children tested in two other tasks (Pino Escobar et al., under review). Online group 2 included 26 additional children in the same age range (M = 4.61 years; SD = 0.51 years, range 4 years - 5.259 males and 17 females) who were tested to increase the sample size of the online study to further test the validity of our online method.

The study was reviewed and approved by the Western Sydney University Human Research Ethics Committee. Informed consent was obtained from all participants' parents in accordance with the approved Ethics Protocol.

### Procedure

The session began by guiding the parent and child through a series of “set-up” slides using the “share screen” function on Zoom. Our set-up slides were adapted from those found in the web resources of the Stanford Social Learning Lab (see Supplementary Figure 2) and began by outlining the study, explaining how long it would take, and what it involved. These slides were crucial in setting expectations for parental involvement and ensuring that the participants' screen was set up correctly.

Expectations for parental involvement were set by instructing the parent about ways in which they could support their child during the study without influencing their child's responses. Suggestions to the parent were statements such as, “I'm not sure, what do you think?,” “Take a guess!.” Parents were asked to stay as neutral as possible throughout the study to reduce the likelihood of their facial reactions or expressions influencing their child's responses. Subsequently, a series of checks regarding participants screen set up were conducted. These checks included a sound test, set-up of full-screen mode, and hiding the pop up of participant and host videos such that only the presentation was visible to child and parent.

Participant set-up took approximately 5 min, after which the eBook and picture-choice task (described below) were presented. At the end of the session, children were asked to rate how much they liked the session by choosing one of three bubbles from small to large, representing little to substantial enjoyment. They were also given the opportunity to ask any questions they may have about the activities they performed during the session, with child-friendly answers provided after each question. Parents were asked whether they experienced or noticed any issues with audio or video quality during the session that might have affected their child's performance, which were noted by the experimenter. No issues were reported for the 41 children included in the current sample. Finally, parents were provided with a verbal debrief of the study.

#### eBook (Learning Phase)

After making sure that the session was correctly set up, the experimenter began recording the Zoom call and proceeded with the task, which comprised a learning phase followed by a test phase. In the learning phase, the experimenter played an audio-visual story which was presented in the form of an eBook that contained novel words embedded within the story.

**Figure 1:**
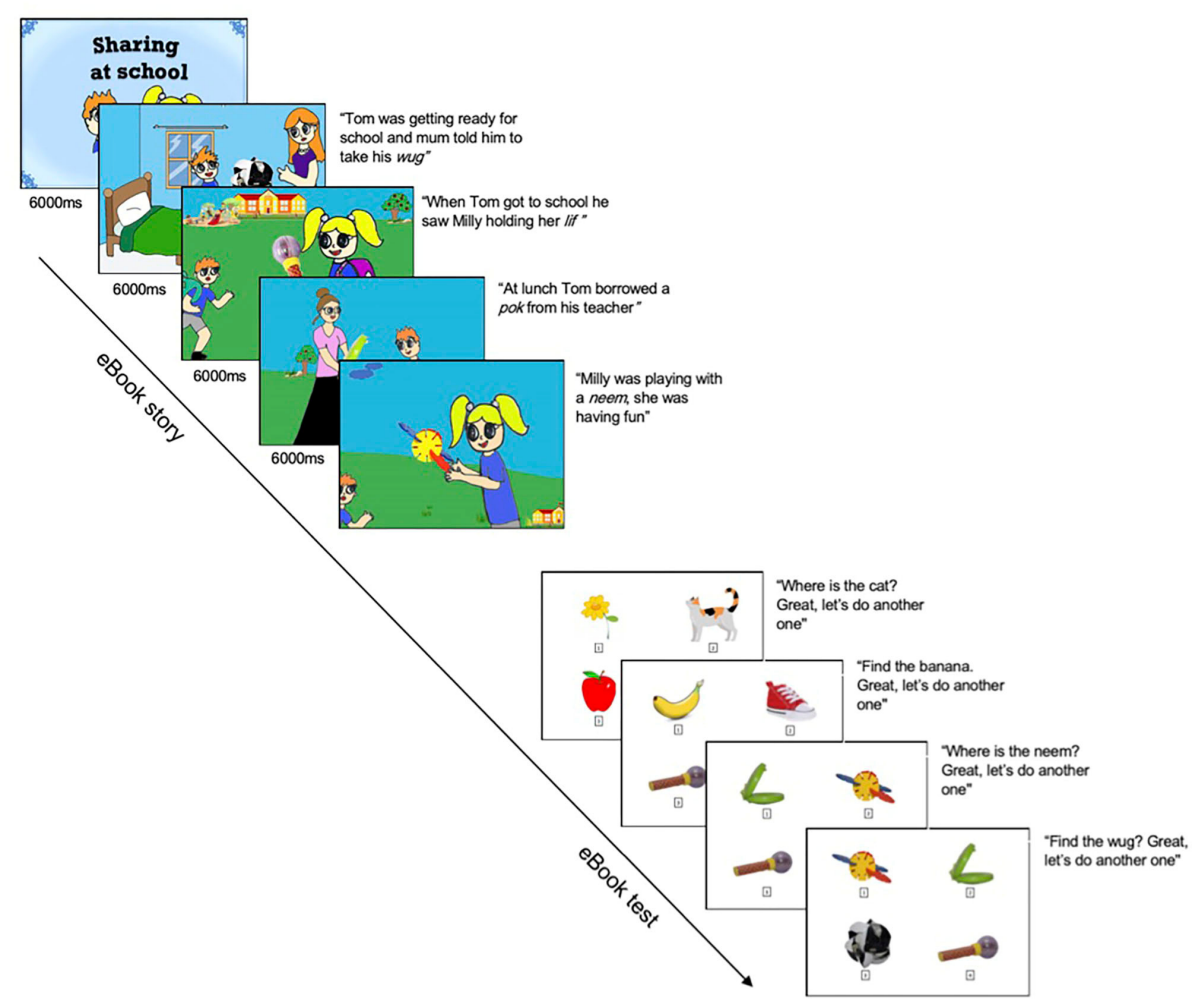
A selection of pages from the learning (eBook) and test (picture-choice task) phases.

The 12-page eBook depicted a story of two children at school sharing four novel objects, examples of these pages are shown in Figure 1. Each page presented colorful, 2D line drawings and included pre-recorded audio from a female native speaker of Australian English. The four novel words and their visual referents were couched in the story among familiar English words and images. The selected novel words WUG, LIF, POK, and NEEM have been used extensively in previous word-learning research with young children (Byers-Heinlein et al., 2013; Kalashnikova et al., 2018). All novel words and corresponding objects were presented three times at various points in the story and children listened to the story twice, and were thus exposed to each novel word-object pairing six times.

The order of the 12 pages of the eBook was fixed, with one word per page and in the same presentation order across participants as follows: WUG, WUG, LIF, WUG, LIF, LIF, POK, POK, NEEM, POK, NEEM, NEEM. PowerPoint was used for the online presentation of the eBook and picture choice test, while Eprime was used for face-to-face presentation. Following Pino Escobar et al. (under review) for both online and in-person versions, each page of the eBook was timed to turn at 6 s after the audio script was played. For a comprehensive list of every adaptation made from the face-to-face testing procedure for the online procedure, please see the Supporting Information.

#### Picture-Choice Task (Test Phase)

Immediately after eBook exposure, the experimenter presented the children with the test phase that consisted of a picture-choice task with 11 trials (3 warm-up trials and 8 test trials). Visual and auditory stimuli were identical across face-to-face and online testing. Warm-up trials, designed to introduce children to the force-choice paradigm, consisted of four pictures and required children to identify a familiar object, for example “Where is the banana?” Test trials (two per novel word) assessed children's memory of the four novel word-object pairs presented in the eBook, and required children to select the referent for a target novel word, for example “Where is the WUG?” The position of the four novel objects on the screen changed in each trial in a pseudorandom order.

For warm-up and test trials, children tested online responded by either verbally indicating which number corresponded to the chosen object or by pointing to the chosen object whilst parents reported their selection. Children tested face-to-face responded by touching the object on the screen (see Supplementary Figure 3 and Supplementary Table 1 for comparisons between the online and face-to-face task). The difference in children's response choices between testing conditions was due to the experimenter's ease of response recording in the online version. A touch screen iPad was used for in-person testing and allowed children to touch the screen to give their responses, while the Zoom environment and the use of different devices in the online condition did not allow for a touch screen option. As well, while it was easy to see where the child pointed during face-to-face testing, this was more difficult online and therefore asking the child to verbalize their choice gave a more accurate indication of their choice should it be difficult to see their pointing on the video chat. Children's age influenced whether they were able to say the number and thus parents' report of their child's choice was a useful measure, especially for young children.

## Results

Enjoyment ratings for the online sessions indicated that the majority of children (*N* = 32) liked it very much as indicated by their choice of the large option, while the remaining children liked the session a reasonable amount, as indicated by their choice of the medium option (*N* = 7) or only a little bit, as indicated by the smallest option (*N* = 2). All 56 children (in both the online and face-to-face groups) responded accurately to the three warm up trials, demonstrating that all children understood the task very well.

### Comparable Face-to-Face and Online Performance

A one-sample *t*-test on the accuracy of children's responses averaged across all test trials was conducted for both Online 1 and Online 2. This indicated that children's performance was significantly above chance for both Online 1, (0.25), M = 0.65, SD = 0.23, *t*(14) = 6.66, *p* < 0.001, and Online 2, (0.25), M = 0.56, SD = 0.29, *t*(25) = 5.62, *p* < 0.001. An identical one-sample *t*-test with the face-to-face sample from Pino Escobar et al. (under review) indicated that these children also performed above chance (0.25) and that their mean accuracy to target word was descriptively very similar to that of the online participants, M = 0.67, SD = 0.24, *t*(14) = 6.61, *p* < 0.001.

**Figure 2:**
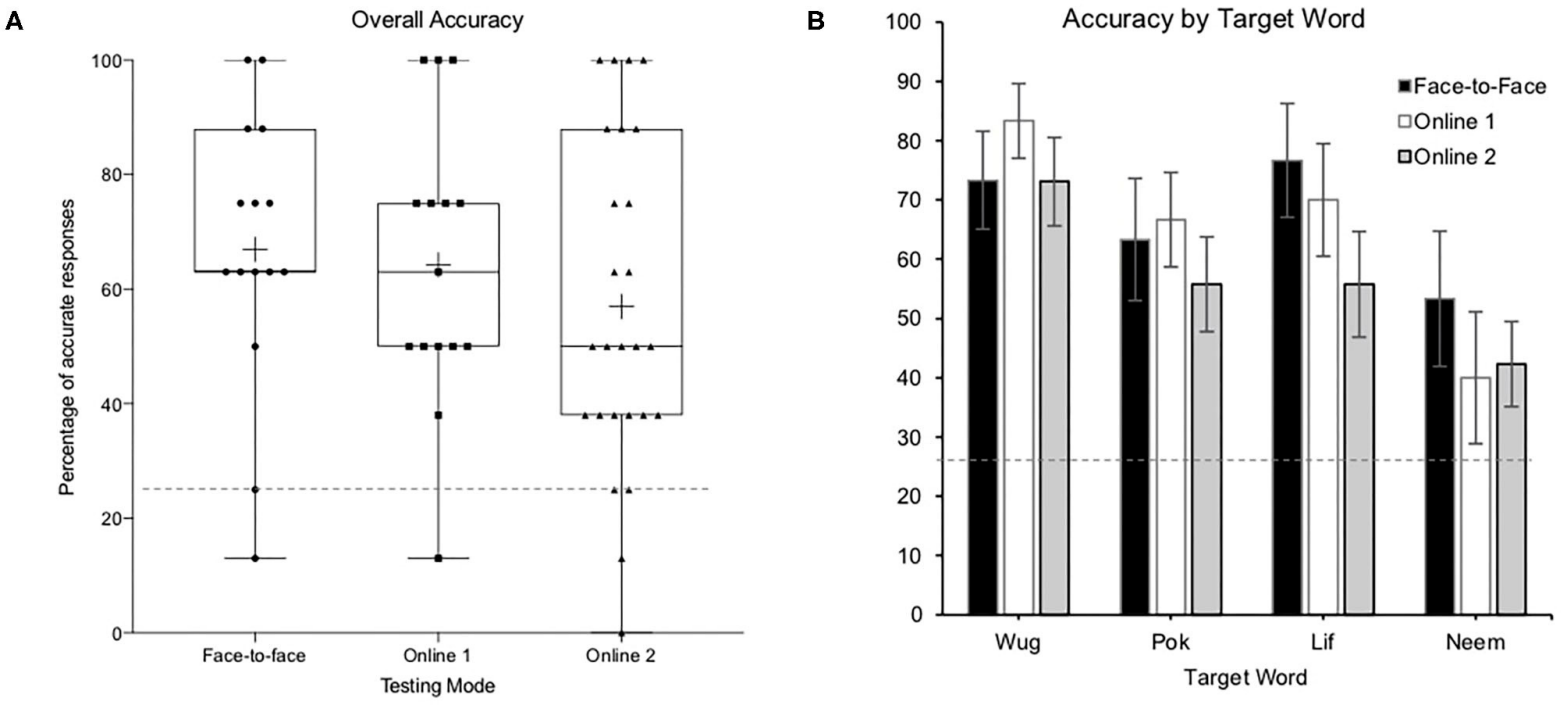
Percentage of children answering correctly **(A)** collapsed across all target words by target word and **(B)** separated by target word. The dotted line presents chance level (25%).

In order to investigate whether children's performance in the face-to-face and online conditions varied based on the target word, we conducted four one-sample *t*-tests per delivery mode. Children, across all three groups, performed significantly above chance for all four target words (Table 1, Figure 2), except for children in Online 1 on the target word NEEM, (0.25), M = 0.40, SD = 0.43, *t*(14) = 1.35, *p* = 0.199.

**Table 1:** Test statistics for each one-sample *t*-tests on target word performance compared to chance (0.25).

	**Face-to-face (*****N*** **= 15)** **Test value** **=** **0.25**				**Online 1 (*****N*** **= 15)** **Test value** **=** **0.25**				**Online 2 (*****N*** **= 26)** **Test value** **=** **0.25**			
	**M (SD)**	***t***	***p***	**95% CI**	**M (SD)**	***t***	***p***	**95% CI**	**M (SD)**	***t***	***p***	**95% CI**
WUG	0.73 (0.32)	5.85	** <0.001**	0.306, 0.661	0.83 (0.24)	9.26	** <0.001**	0.306, 0.661	0.73 (0.38)	6.45	** <0.001**	0.306, 0.661
POK	0.63 (0.40)	3.72	**0.002**	0.162, 0.605	0.67 (0.31)	5.23	** <0.001**	0.162, 0.605	0.56 (0.41)	3.85	**0.001**	0.162, 0.605
LIF	0.77 (0.37)	5.39	** <0.001**	0.311, 0.723	0.70 (0.37)	4.73	** <0.001**	0.311, 0.723	0.56 (0.45)	3.45	**0.002**	0.311, 0.723
NEEM	0.53 (0.44)	2.48	**0.026**	0.039, 0.528	0.40 (0.43)	1.35	0.199	0.039, 0.528	0.42 (0.37)	2.41	**0.024**	0.039, 0.528

*Bold values indicate p < 0.05*.

### Differential Word-by-Word Performance for all Children

In order to test whether performance differences revealed with *t*-tests were due to testing conditions or to the words presented to all children, we analyzed children's word-learning accuracy with generalized linear mixed models on a binomial distribution using the GLIMMIX procedure in SAS 9.3 (Stroup, 2016) (results summarized in Table 2). The following full factorial model was analyzed: Testing mode (face-to-face vs. online 1 vs. online 2) × Target Word (WUG, POK, LIF, or NEEM) × Age (mean-centered continuous variable ranging from 4.00 to 5.40 years). In addition, to examine whether accuracy varied as a function of Testing Mode by Target Word, this two-way interaction term was included. A random intercept to account for individual participant differences was also included. Finally, gender (male vs. female) was included as a control variable.

**Table 2:** Model 1.

**Variable**	***b***	***se***	***p***
**Fixed effects**			
Intercept	1.39	0.44	0.003
Testing mode	*F* = 0.33		0.716
Target word	*F =* 8.87		** <0.001**
Age (years)	0.45	0.48	0.36
Testing mode × Target word	*F =* 0.71		0.064
**Random effects**			
Intercept	1.35	0.49	**0.003**
AIC	556.13		

*Fixed effects for children's accuracy. Note. F values are indicated wherever it was not possible to obtain a single b value to summarize the effect. This was the case for all effects involving Target Word, as this IV contained four categorical levels. Bold values indicate p < 0.05*.

The full factorial model revealed a significant effect of target word, *F* = 8.87, *p* = <0.001 such that children, regardless of their testing condition they were in, performed better on some target words than others. There was no other significant fixed effect and no significant two-way interaction, indicating that children in all testing conditions learned some words better than others. Pairwise comparisons were conducted to identify the differential levels of accuracy for the four words. Children had lower word learning accuracy for NEEM than WUG, *b* = 1.76, se = 0.35, *p* < 001, 95% CI [(0.82, 2.69), LIF, *b* = 0.1.19, se = 0.33, *p* = 0.002, 95% CI (0.31, 2.09), or POK, *b* = 0.87, se = 0.32, *p* = 0.043, 95% CI (0.02, 1.72)].

## Discussion

The current study highlights that children's performance is comparable across face-to-face and online testing modes. Furthermore, we illustrated successful child testing with preschool aged children in an interactive experiment as well as successful word learning from an eBook. Thus, the crucial finding is that our online paradigm yielded no hindrance to children's learning, complementing the works of Sheskin and Keil (2018) and Kapitány et al. (2020). We believe that this is due to a key component of our paradigm, namely an emphasis on positive and clear communication with parents during recruitment and the online testing session. Specifically, we found parents to be interested in and grateful for the opportunity to participate in research in the comfort of their homes, avoiding the hassle and time involved in a laboratory visit, and without any potential risks to their health and safety. Likely due to the convenience of participating from home, we also found parents to be receptive to and successful in following the instructions provided regarding their involvement during the study. Further, children were enthusiastic about participating in the study, were engaged throughout and reported high levels of enjoyment. This high level of engagement and enthusiasm may be due to the stay-at-home advice during the global pandemic. However, the children included in the online condition were tested in Australia where no mandate for preschool children to stay at home had been provided, with preschools remaining open and with parents encouraged to send their children to preschool as per their usual arrangements.

Despite this validation success, it is worth mentioning that the face-to-face condition included only 15 children, as those were the children tested for the face-to-face eBook condition in a previous study (Pino Escobar et al., under review). Unfortunately, due to COVID-19, face-to-face testing to increase this sample size has not been possible. Although similar sample sizes have been reported in previous, similar studies (Giezen et al., 2016; Pino Escobar et al., 2018; Junttila and Ylinen, 2020), we acknowledge this as a potential limitation, which can be overcome with extra data collection when the current pandemic allows or by using a Bayesian approach when comparing in-person and online results [see for instance Escudero et al. (2020) and Escudero et al. (2021)].

In addition to validating the online delivery of a word learning task typically administered in person, we also found that children's word learning was highly successful, replicating the findings of much previous research (Reich et al., 2016). We found children learned almost all words above chance with only one word (NEEM) learnt at chance level for the Online 1 group only. NEEM was also the word learnt poorest out of all four words when performance was averaged across all three groups. We speculate that order effects may have driven this particular effect, as the word NEEM was presented in the final slides of the eBook (see Learning Phase in the Method section). Research on word learning from storybooks suggests that children are increasingly less successful at acquiring novel words as the number of new tokens increases (Flack et al., 2018). The number of novel words children are capable of learning from this particular eBook may be bordering on four, with the effect of NEEM least powerful relative to the three other novel words presented earlier. Thus, although some variation was identified on the performance of some words, the online component of this study behaves identically to the face-to-face component and additionally replicates the variation observed in other studies supporting the notion that word learning research can be successfully deployed online.

As experimental scientists working with young children, we are faced with an uncertain future and unprecedented demand has arisen for the transition to online developmental research. The current study heeds this call and provides evidence that online child-based experimentation is possible, successful and comparable to face-to-face developmental science. Importantly, we believe that providing parents with the option for research participation in an online format from the comfort of their home is more inclusive, reaching a wider community. This is due to the increased availability of broadband technology and remote learning devices such as iPads or laptops for families of diverse socio-economic status, for whom travel to a university laboratory or access to paid, private pre-schooling is not possible (cf. Day et al., 2021).

We provide a framework and resources through which a broad range of developmental tasks can be administered online. Moving forward, amidst the COVID-19 pandemic and beyond when the world begins to open again, the use of online developmental science will continue to be a valuable component of our research. We propose that online research should not be considered a solution in the interim, but rather a new and effective tool for upscaling our research and accessing wider and more diverse populations additionally lending itself to longitudinal designs and opening up avenues to different research questions. Further research is certainly needed to document the differences between face-to-face and online testing for a broad range of developmental phenomena, ages and across a spectrum of development diagnoses and delays in addition to typically developing children. However, the resources and evidence provided here constitute the foundational support for accomplishing these bigger goals.

## Data Availability Statement

The raw data supporting the conclusions of this article will be made available by the authors, without undue reservation.

## Ethics Statement

The studies involving human participants were reviewed and approved by Western Sydney University Human Research Ethics Committee. Written informed consent to participate in this study was provided by the participants' legal guardian/next of kin.

## Author Contributions

PE, GP, CC, and KS: conceptualization, method, and manuscript writing. GP, CC, and KS: literature review, data collection, and curation. PE, GP, and KS: statistical analysis. PE: project sourcing, leadership, and supervision. All authors contributed to the article and approved the submitted version.

## Conflict of Interest

The authors declare that the research was conducted in the absence of any commercial or financial relationships that could be construed as a potential conflict of interest.
