# Supplementary material for: Four-Year-Old's Online Versus Face-to-Face Word Learning via eBooks
Source: Front Psychol. 2021 Mar 12;12:610975. doi: 10.3389/fpsyg.2021.610975 (PMC7994518; doi:10.3389/fpsyg.2021.610975)
Supplement: Supplementary file 1 [file Table_1.PDF]

Supplementary Table 1: Comparison Online versus Face-to-Face Methods

**Task stages**	**Face-to-face**	**Online**	**Observation**
Learning phase	The audio narrative on each slide lasted 6000 ms.<br><br>After each slide, a red arrow appeared on the inferior-right-hand side of the screen for 3000 ms. Children had the option to touch the arrow to progress to the next slide before it did so automatically (after 3000 ms).<br><br>Children listened to the story twice, and in total were exposed to each novel word-object pair six times.	The audio narrative on each slide lasted 6000 ms.<br><br>There was a pause of 500 ms between each slide before it automatically progressed to the next slide. Children were not shown the red arrow and were not given the opportunity to progress to the next slide.<br><br>Children listened to the story twice, and in total were exposed to each word-object pair six times.	Same<br><br>Variation<br><br>Same
Familiarisation and testing phases	Explicit forced-choice receptive task with three familiarisation trials and eight test trials. Each novel word-object pair tested twice.<br><br>Each trial presented four images positioned to the superior-left, inferior left, superior-right and inferior-right of the screen.<br><br>Children responded to trials by touching the screen. After doing so, it automatically progressed to the next trial.	Explicit forced-choice receptive task with three familiarisation trials and eight test trials. Each novel word-object pair tested twice.<br><br>Each trial presented four images positioned to the superior-left, inferior left, superior-right and inferior-right of the screen. Images were numbered 1 to 4.<br><br>Children responded in one of two ways. Either they pointed to their selection and their parent reported the number of that image Or the child verbally reported the number of the image themselves. After each response the experimenter progressed to the next trial.	Same<br><br>Variation<br><br>Variation
