# Supplementary figures and images for: Four-Year-Old's Online Versus Face-to-Face Word Learning via eBooks
Source: Front Psychol. 2021 Mar 12;12:610975. doi: 10.3389/fpsyg.2021.610975 (PMC7994518; doi:10.3389/fpsyg.2021.610975)

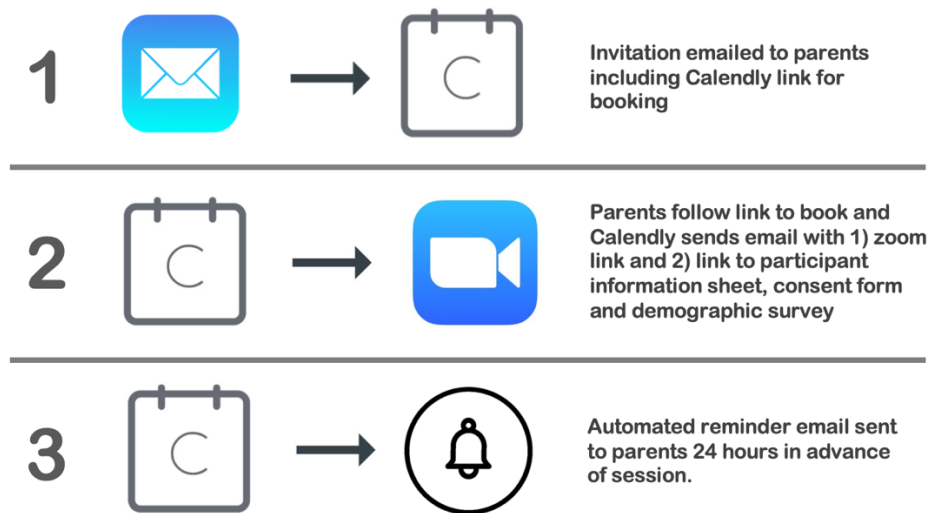

Supplementary Figure 1. A visual overview of the recruitment workflow used in this study.

Supplement: Supplementary file 2 [file Image_1.PDF]
