# Supplementary material for: Four-Year-Old's Online Versus Face-to-Face Word Learning via eBooks
Source: Front Psychol. 2021 Mar 12;12:610975. doi: 10.3389/fpsyg.2021.610975 (PMC7994518; doi:10.3389/fpsyg.2021.610975)
Supplement: Supplementary file 3 [file Image_2.PDF]

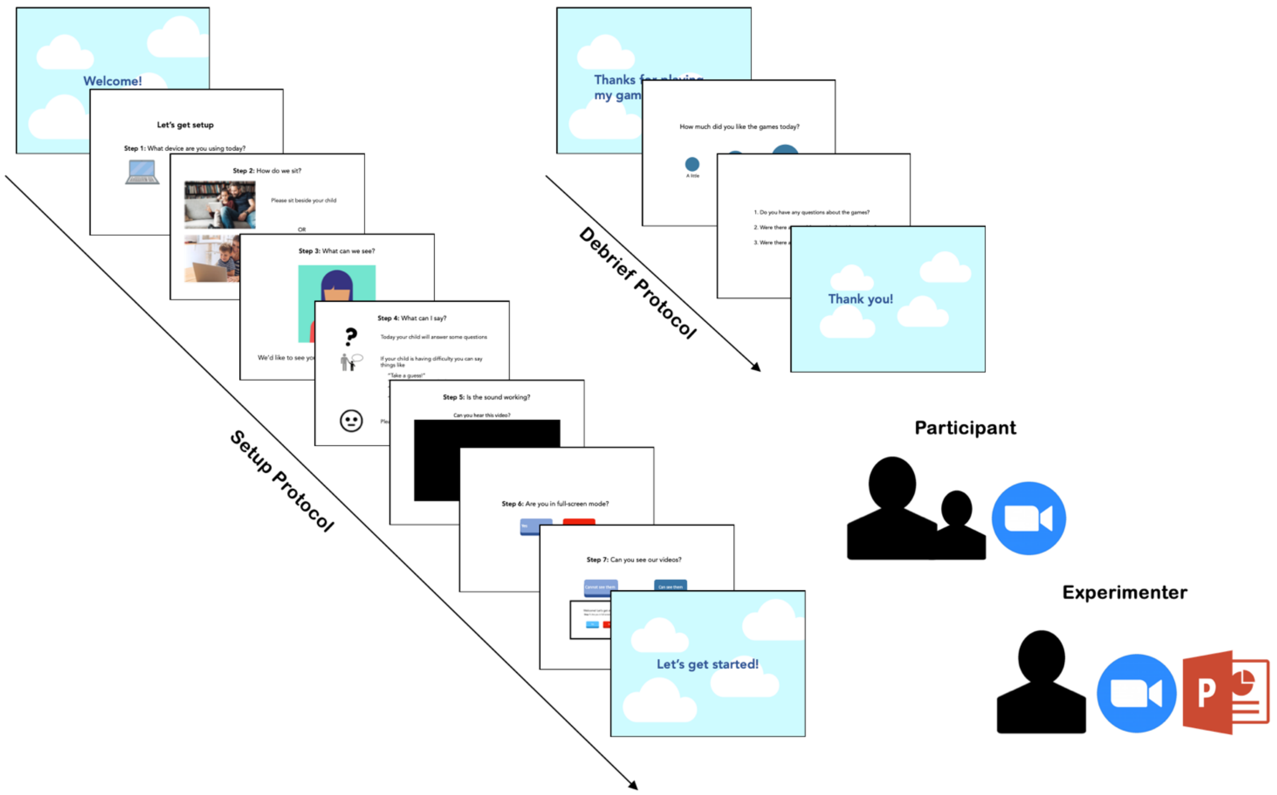


Supplementary Figure 2: Representation of the study procedure, delivered using Zoom and Powerpoint.
