# Supplementary material for: Four-Year-Old's Online Versus Face-to-Face Word Learning via eBooks
Source: Front Psychol. 2021 Mar 12;12:610975. doi: 10.3389/fpsyg.2021.610975 (PMC7994518; doi:10.3389/fpsyg.2021.610975)
Supplement: Supplementary file 4 [file Image_3.PDF]

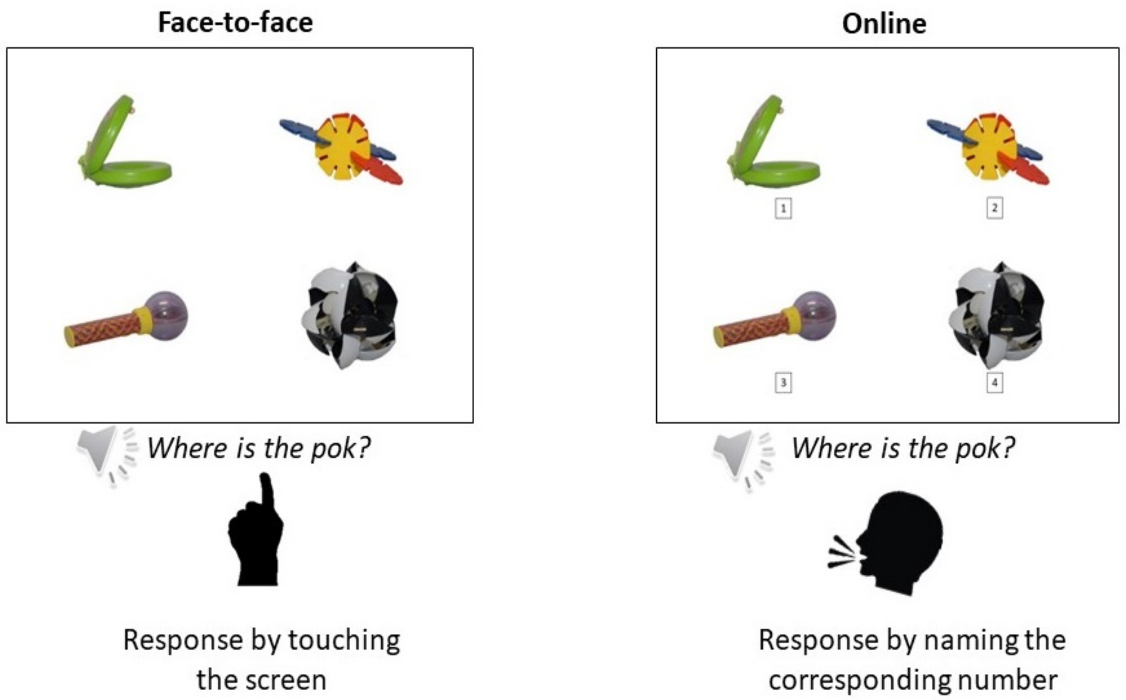

Supplementary Figure 3. Example of a testing trial in face-to-face testing (left) and the online adaptation (right).
